# Prediction of Liver-Related Events Using Fibroscan in Chronic Hepatitis B Patients Showing Advanced Liver Fibrosis

**DOI:** 10.1371/journal.pone.0036676

**Published:** 2012-05-04

**Authors:** Seung Up Kim, Ji Hoon Lee, Do Young Kim, Sang Hoon Ahn, Kyu Sik Jung, Eun Hee Choi, Young Nyun Park, Kwang-Hyub Han, Chae Yoon Chon, Jun Yong Park

**Affiliations:** 1 Department of Internal Medicine, Yonsei University College of Medicine, Seoul, Korea; 2 Institute of Gastroenterology, Yonsei University College of Medicine, Seoul, Korea; 3 Department of Biostatistics, Yonsei University College of Medicine, Seoul, Korea; 4 Department of Pathology, Yonsei University College of Medicine, Seoul, Korea; 5 Liver Cirrhosis Clinical Research Center, Seoul, Korea; 6 Brain Korea 21 Project of Medical Science, Seoul, Korea; The University of Hong Kong, Hong Kong

## Abstract

**Background:**

Liver stiffness measurement (LSM) using transient elastography (FibroScan®) can assess liver fibrosis noninvasively. This study investigated whether LSM can predict the development of liver-related events (LREs) in chronic hepatitis B (CHB) patients showing histologically advanced liver fibrosis.

**Methods:**

Between March 2006 and April 2010, 128 CHB patients with who underwent LSM and liver biopsy (LB) before starting nucleot(s)ide analogues and showed histologically advanced fibrosis (≥F3) with a high viral loads [HBV DNA ≥2,000 IU/mL] were enrolled. All patients were followed regularly to detect LRE development, including hepatic decompensation (variceal bleeding, ascites, hepatic encephalopathy, spontaneous bacterial peritonitis, hepatorenal syndrome) and hepatocellular carcinoma (HCC).

**Results:**

The mean age of the patient (72 men, 56 women) was 52.2 years. During the median follow-up period [median 27.8 (12.6–61.6) months], LREs developed in 19 (14.8%) patients (five with hepatic decompensation, 13 with HCC, one with both). Together with age, multivariate analysis identified LSM as an independent predictor of LRE development [*P*<0.044; hazard ratio (HR), 1.038; 95% confidence interval (CI), 1.002–1.081]. When the study population was stratified into two groups using the optimal cutoff value (19 kPa), which maximized the sum of sensitivity (61.1%) and specificity (86.2%) from a time-dependent receiver operating characteristic curve, patients with LSM>19 kPa were at significantly greater risk than those with LSM≤19 kPa for LRE development (HR, 7.176; 95% CI, 2.257–22.812; *P* = 0.001).

**Conclusion:**

LSM can be a useful predictor of LRE development in CHB patients showing histologically advanced liver fibrosis.

## Introduction

Chronic infection with hepatotropic viruses is the main cause of chronic liver disease (CLD) worldwide, and hepatitis B virus (HBV) is predominant in the Far East [1], [2]. Approximately 10–20% of patients with chronic hepatitis B (CHB) infection have liver cirrhosis at first presentation, and an additional 20–30% of patients will eventually develop this condition and its complications within one or more decades [3], [4]. Previous studies indicated an annual risk of developing hepatocellular carcinoma (HCC) of 1–6%, and a similar or higher risk of hepatic decompensation after the development of cirrhosis [5], [6]. Although antiviral treatment using nucleot(s)ide analogues (NUCs) suppresses HBV effectively [7], liver-related events (LREs) including hepatic decompensation, HCC, and liver-related death still occur and remain an important watershed in the management algorithm of patients with CHB.

Because LREs usually develop in patients with advanced liver fibrosis and cirrhosis, the early detection of advanced liver fibrosis and cirrhosis and the assessment of their severity for the design of optimal surveillance and intervention strategies are important. Although liver biopsy (LB) has been the gold standard for assessing liver fibrosis to date [8], it is prone to sampling error and interpretational variability [9]. Recently, liver stiffness measurement (LSM) using transient elastography (FibroScan®) has been introduced for assessing liver fibrosis with accurate, reproducible, and reliable results [10], [11]. Furthermore, because LSM can be expressed numerically as a continuous variable, clinicians can grade the degree of liver fibrosis, even in patients with cirrhosis, and assess the risk of developing liver-related complications and HCC [12]–[14]. Thus, we hypothesized that LSM could predict the development of LREs in CHB patients who were receiving antiviral treatment using NUCs due to histologically advanced liver fibrosis or cirrhosis with a high viral load.

Previous cross-sectional studies have reported an association between LSM and the presence of liver-related complications or HCC in patients with CLD [12], [13], [15], [16]. However, few prospective longitudinal studies have investigated the role of LSM as a predictor of LRE development in patients with advanced liver fibrosis. Thus, we evaluated the usefulness of LSM in assessing the risk of LRE development in CHB patients showing histologically advanced liver fibrosis with a high viral load.

## Methods

### Patients

Between March 2006 and April 2010, a total of 178 NUC-naïve CHB patients underwent LB to assess the degree of liver fibrosis and necroinflammation before starting antiviral treatment at Severance Hospital, Yonsei University College of Medicine, Seoul, Korea. CHB was defined as the persistent presence of serum hepatitis B surface antigen (HBsAg) for more than 6 months and HBV DNA positivity on a polymerase chain reaction (PCR) assay. Patients who provided written informed consent and received LB and LSM were consecutively enrolled in this prospective study. The study protocol conformed to the ethical guidelines of the 1975 Declaration of Helsinki and was approved by the institutional review board of Severance Hospital.

Exclusion criteria were as follows: 1) LSM failure (no valid shots; *n* = 0), 2) invalid LSM [defined as an interquartile range (IQR) to median value ratio (IQR/M) >0.3, success rate <60%, or <10 valid measurements; *n* = 6] [17], 3) a history of hepatic decompensation or antiviral treatment (*n* = 0), 4) co-infection with hepatitis C, hepatitis D, or HIV (*n* = 1), 5) heavy alcohol consumption (>30 g/day for >5 years; *n* = 4), 6) right-sided heart failure, ascites, or pregnancy (*n* = 0), 7) F0–2 fibrosis stage on LB (*n* = 20), 8) low viral load (<2,000 IU/mL; *n* = 7), 9) LB specimen shorter than 15 mm (*n* = 9), and 10) follow-up loss (*n* = 3) (**Figure S1**).

A total of 50 patients were excluded; the remaining 128 CHB patients showing advanced (≥F3) liver fibrosis on LB with a high viral load (HBV DNA ≥2,000 IU/L) were selected for the final statistical analysis (**Figure S1**).

### Laboratory tests

On the same day as LB and LSM, blood parameters including serum albumin, total bilirubin, alanine aminotransferase (ALT), prothrombin time, platelet count, and alpha-fetoprotein (AFP) were recorded. HBsAg and hepatitis B e antigen (HBeAg) were measured using standard enzyme-linked immunosorbent assays (Abbott Diagnostics, Abbott Park, IL, USA). HBV DNA levels were measured by quantitative PCR assay (Amplicor HBV Monitor Test; Roche Diagnostics, Basel, Switzerland) with a detection limit of 12 IU/mL. The upper normal range of ALT was 40 IU/L.

### Liver stiffness measurement

On the same day as LB, LSM was performed on the right lobe of the liver through the intercostal spaces on patients lying in the dorsal decubitus position with the right arm in maximal abduction [17]. One experienced technician (>10,000 examinations) who was blinded to the patients' clinical data performed all LSMs. The success rate was calculated by dividing the number of valid measurements by the total number of measurements. IQR was defined as an index of intrinsic variability of LSM corresponding to the interval of LSM results containing 50% of the valid measurements between the 25th and 75th percentiles [14]. LSM scores are expressed as kilopascals (kPa). When LSM showed an IQR/M >0.3, success rate <60%, or <10 valid measurements, it was regarded as invalid and was excluded from the final analysis.

### Liver biopsy and liver histology evaluation

LB specimens were fixed in formalin and embedded in paraffin. Four-micrometer-thick sections were stained with hematoxylin & eosin and Masson's trichrome. All liver tissue samples were evaluated by an experienced hepatopathologist who was blinded to the clinical data of the study population, including LSM results. Liver fibrosis and necroinflammation were evaluated semiquantitatively according to the Batts scoring system [18]. Fibrosis was staged on a 0–4 scale: F0, no fibrosis; F1, portal fibrosis; F2, periportal fibrosis; F3, septal fibrosis; and F4, cirrhosis. The activity grade referred to the degree of hepatocellular necroinflammatory activity: A0, no activity; A1, minimal; A2, mild; A3, moderate; and A4, severe activity. Steatosis in the liver specimen was graded on a four-point scale: S0 (non-significant, <5%), S1 (mild, 5–33%), S2 (moderate, 34–66%) and S3 (severe, ≥66% of hepatocytes with fat deposits) [19]. LB specimens shorter than 15 mm were considered ineligible and excluded from the final analysis. The median length of LB specimens was 17 mm (range, 15–23 mm).

### Follow up

All patients underwent LB and LSM and were screened ultrasonographically for HCC at their initial visit. NUCs, such as lamivudine and entecavir, were chosen non-randomly after enrollment according to the patient's economic status and physicians' judgment, and an HCC surveillance program was initiated in all patients. Patients were followed up with AFP and ultrasonography every 3 or 6 months. Furthermore, patients were assessed at baseline and every 3 months thereafter for clinical evidence of hepatic decompensation, including variceal bleeding, ascites, hepatic encephalopathy (HE), spontaneous bacterial peritonitis (SBP), and hepatorenal syndrome (HRS). At the end of the follow-up period (March 2011), one patient had died due to HCC and another had undergone liver transplantation after ascitic decompensation.

### Primary endpoint

The primary endpoint was the development of LREs, including hepatic decompensation (variceal bleeding, ascites, HE, SBP, and HRS) and HCC.

### Definition of hepatic decompensation

Variceal bleeding was diagnosed endoscopically if hemorrhage from the dilated veins in the distal esophagus or proximal stomach caused by elevated pressure in the portal venous system was noted [20]. Ascites was diagnosed by imaging, such as computed tomography (CT) or ultrasonography, if fluid collection within the abdominal cavity associated with cirrhosis was noted [21]. HE was diagnosed if confusion, altered level of consciousness, and coma developed as a result of liver failure, after the exclusion of known brain disease [22]. SBP was defined as an ascitic fluid infection without an intra-abdominal, surgically treatable source [23]. Diagnosis was established by a positive ascitic fluid bacterial culture and an elevated ascitic fluid absolute polymorphonuclear leukocyte count (≥250 cells/mm3). HRS was diagnosed when acute renal failure developed in association with advanced chronic liver disease, after the exclusion of other causes of renal failure [24].

### Diagnosis of HCC

HCC was diagnosed based on the guidelines of the American Association for the Study of Liver Diseases (AASLD) [25]. Briefly, patients were diagnosed with HCC if they had a tumor with a maximum diameter >2 cm, features typical of HCC on dynamic CT (hyperattenuation in the arterial phase and early washout in the portal phase), and AFP >200 ng/mL. If the maximum diameter of the tumor was 1–2 cm, dynamic CT and magnetic resonance imaging were performed and HCC was diagnosed if coincidental typical features of HCC were noted. If the tumor did not satisfy the above criteria, a biopsy was performed. When the tumor was <1 cm, ultrasonographic examination was repeated after 3 months.

### Statistical analyses

Data are expressed as the mean ± standard deviation (SD), median (range), or *n* (%), as appropriate. Baseline characteristics of patients with and without LRE development were compared using the chi-squared and Fisher's exact tests. To identify independent predictors of LRE development, univariate and subsequent multivariate Cox proportional hazard regression analyses were used. Hazard ratios (HRs) and corresponding 95% confidence intervals (CIs) are indicated. Time-dependent receiver operating characteristic (ROC) curves and areas under the ROC (AUROC) were used to calculate the optimal LSM cutoff value for the prediction of LRE development, which maximized the sum of sensitivity and specificity. The annual incidence rates of HCC were expressed in person-years. The cumulative incidence rates of HCC were calculated using the Kaplan–Meier method. A *P* value<0.05 on a two-tailed test was considered statistically significant. Statistical analyses were performed using SPSS software (ver. 18.0; SPSS Inc., Chicago, IL, USA).

## Results

### Baseline characteristics

The baseline characteristics of 128 patients at enrollment are summarized in **Table 1**. The mean age of the patients (72 men and 56 women) was 52.2 years. All patients with cirrhosis showed preserved liver function of Child–Pugh class A. The mean body mass index (BMI) and ALT were 24.0 kg/m^2^ and 44.4 IU/L, respectively, and the median LSM value was 12.9 kPa.

**Table 1:** Baseline Characteristics (n = 128).

Variables	Values
**Demographic data**	
Age (years)	52.2±9.3
Male	72 (56.3)
Diabetes mellitus	11 (8.6)
Body mass index (kg/m^2^)	24.0±2.9
**Laboratory data**	
Serum albumin (g/dL)	4.2±0.5
Total bilirubin (mg/dL)	0.8±0.3
Alanine aminotransferase (IU/L)	44.4±21.6
Prothrombin time (%)	90.9±9.8
Platelet count (10^9^/L)	148.5±48.4
Alpha-fetoprotein (ng/mL)	4.7 (0.8–50.6)
**Histological data**	
Fibrosis stage, 3/4	18 (14.1)/110 (85.9)
Activity grade, 1–2/3–4	97 (75.8)/31 (24.2)
**Liver stiffness measurement**	
LSM values (kPa)	12.9 (4.4–57.1)
Success rate (%)	100 (63–100)
Interquartile range/median value (kPa)	0.15 (0.01–0.30)
**Antiviral agent**	
Lamivudine	49 (38.3)
Entecavir	79 (61.7)

Variables are expressed as mean ± SD, median (range), or n (%).

HBeAg, hepatitis B e antigen; LSM, liver stiffness measurement; kPa, kilopascal.

F3 and F4 fibrosis stages were noted in 18 (14.1%) and 110 (85.9%) patients, respectively, and most patients (*n* = 97, 75.8%) had a necroinflammatory activity grade of 1–2 (**Table 1**). S0–1 steatosis was identified in 127 (99.2%) patients and S2 in one (0.8%), whereas none showed S3 steatosis.

### LRE development and comparisons between patients with and without LREs

**Figure 1:**
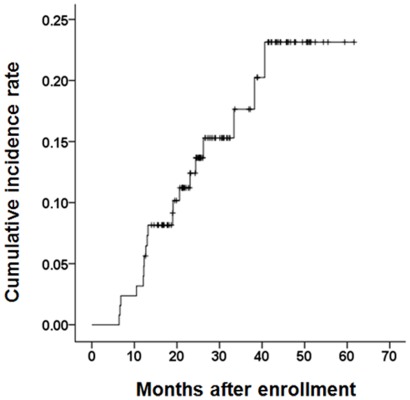
The cumulative incidence rates of LREs (Kaplan-Meier plot). The cumulative incidence rates of LREs at 1, 2, and 3 years were 3.1%, 11.7%, and 16.2%, respectively. LRE, liver-related event.

During the follow-up period [median, 27.8 (range, 12.6–61.6) months] constituting a total of 297 person-years, LREs developed in 19 (14.8%) patients (6.4/100 person-years; five cases with decompensation, 13 with HCC, and one with both decompensation and HCC; **Table 2**). The six cases of hepatic decompensation included variceal bleeding in two patients, ascites development in two, and HE in two. SBP and HRS did not develop during the follow-up period. The cumulative incidence rates of LREs at 1, 2, and 3 years were 3.1%, 11.7%, and 16.2%, respectively (**Figure 1**). The incidence rate of HCC and hepatic decompensation was 4.7/100 and 2.0/100 person-years, respectively.

**Table 2:** Comparison Between Patients with and without LRE Development.

Variables	Patients with LRE development (n = 19, 14.8%)	Patients without LRE development (n = 109, 85.2%)	*P* value
**Demographic data**			
Age (years)	56.2±6.5	51.5±9.6	0.043
Male	12 (63.2)	60 (55.0)	0.511
Diabetes mellitus	3 (15.8)	8 (7.3)	0.210
Body mass index (kg/m^2^)	24.1±2.9	23.9±2.9	0.794
HBeAg positivity	12 (63.2)	58 (53.2)	0.422
HBV DNA (log_10_ IU/mL)	5.7±1.1	5.6±1.2	0.610
**Laboratory data**			
Serum albumin (g/dL)	3.9±0.6	4.3±0.4	0.003
Total bilirubin (mg/dL)	0.9±0.3	0.8±0.3	0.181
Alanine aminotransferase (IU/L)	39.4±16.0	45.3±22.4	0.279
Prothrombin time (%)	85.1±12.7	91.9±9.0	0.037
Platelet count (10^9^/L)	124.7±37.4	152.7±49.0	0.019
Alpha-fetoprotein (ng/mL)	6.7 (2.4–38.2)	4.4 (0.8–50.6)	0.020
**Histological data**			
Fibrosis stage, 3/4	4 (21.1)/15 (78.9)	14 (12.8)/95 (87.2)	0.342
Activity grade, 1–2/3–4	12 (63.2)/7 (36.8)	85 (78.0)/24 (22.0)	0.164
**Liver stiffness measurement**			
LSM values (kPa)	21.1 (7.8–57.1)	11.8 (4.4–48.0)	0.011
**Antiviral treatment**			
Antiviral agent			0.515
Lamivudine	6 (31.6)	43 (39.4)	
Entecavir	13 (68.4)	66 (60.6)	
HBV DNA negativity			
At 3 months	10 (52.6)	67 (61.5)	0.468
At 6 months	13 (68.4)	80 (73.4)	0.654
At 12 months	15 (78.9)	92 (84.4)	0.515
YMDD mutation	2(10.5)	15(13.8)	1.000

Variables are expressed as mean ± SD, median (range), or n (%).

LRE, liver-related event; HBeAg, hepatitis B e antigen; LSM, liver stiffness measurement; kPa, kilopascal.

When we compared the baseline characteristics of patients with and without LRE development, serum albumin, prothrombin time, and platelet count were significantly higher in patients without LREs, whereas age, AFP, and LSM values were significantly higher among those with LRE development (all *P*<0.05; **Table 2**). No significant difference was observed in the proportion of fibrosis stage, activity grade, or steatosis between patients with and without LRE development (all *P*>0.05; **Table 2**).

### Antiviral treatment

All patients received antiviral treatment with either lamivudine (*n* = 49, 38.3%) or entecavir (*n* = 79, 61.7%; **Table 1**). Baseline characteristics, including demographic, laboratory, and histologic data, did not differ between patients who received lamivudine and those who received entecavir (all *P*>0.05). Furthermore, the treatment period until the end of follow-up was similar (median 29.1 months for lamivudine *vs*. 27.2 months for entecavir; *P* = 0.785). HBV DNA negativity at 3, 6, and 12 months of antiviral treatment, type of antiviral agent, and the development of the YMDD mutation did not influence LRE development (all *P*>0.05; **Table 2**).

Six (12.2%) of the 49 patients treated with lamivudine and 13 (16.5%) patients treated with entecavir developed LREs (*P* = 0.614). During antiviral treatment, the YMDD mutation developed in 17 (13.3%) patients who received lamivudine after a median of 18.5 months; however, no genotypic antiviral resistance was identified in patients treated with entecavir. Of the 17 patients with the YMDD mutation, two experienced LRE development (HCC). Add-on treatment with adefovir was administered to all patients with the YMDD mutation.

### Independent risk factors for LRE development

**Figure 2:**
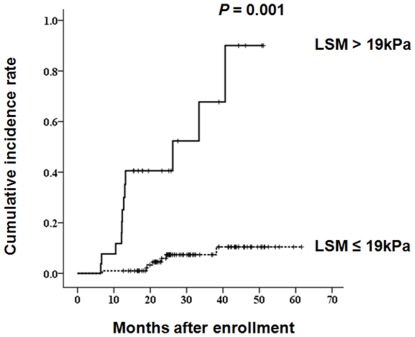
Cumulative incidence rates of LREs based on stratified LSM values (Kaplan-Meier plot). Patients with LSM value >19 kPa were at a significantly greater risk of LREs development with a hazard ratio of 7.176 [95% confidence interval, 2.257–22.812; *P* = 0.001], as compared to those with LSM value ≤19 kPa. LSM, liver stiffness measurement; kPa, kilopascal. LRE, liver-related event.

Together with age, multivariate analysis identified LSM as an independent predictor of LRE development (*P* = 0.044; HR, 1.038; 95% CI, 1.002–1.081; **Table 3**). When we used a time-dependent ROC curve analysis to identify the optimal LSM cutoff values for stratifying our study population into two groups, 19 kPa showed the greatest accuracy (AUROC, 0.722; 95% CI, 0.582–0.864; *P* = 0.003; sensitivity, 61.1%; specificity, 86.2%). Twenty-seven patients with LSM values >19 kPa were found to be at a significantly greater risk of LRE development (HR, 7.176; 95% CI, 2.257–22.812; *P* = 0.001) in comparison with 101 patients with LSM values ≤19 kPa (**Figure 2**).

**Table 3:** Independent Risk Factors for LRE Development.

Variables	Univariate	Multivariate	
	*P* value	Hazard ratio (95% CI)	*P* value
**Demographic data**			
Age	0.045	1.083 (1.008–1.164)	0.030
Male	0.421		
Diabetes mellitus	0.192		
Body mass index	0.667		
**Laboratory data**			
Serum albumin	0.002	0.549 (0.168–1.794)	0.321
Total bilirubin	0.188		
Alanine aminotransferase	0.321		
Prothrombin time	0.005	0.979 (0.935–1.025)	0.363
Platelet count	0.015	0.996 (0.983–1.009)	0.581
Alpha-fetoprotein	0.041	0.984 (0.907–1.067)	0.694
HBeAg positivity	0.517		
HBV DNA	0.623		
HBV DNA negativity at 3 months	0.750		
**Liver biopsy data**			
Fibrosis stage, 3/4	0.352		
Activity grade, 1–2/3–4	0.208		
**Liver stiffness measurement**	<0.001	1.038 (1.002–1.081)	0.044
**Antiviral treatment**			
Lamivudine vs. entecavir	0.442		
HBV DNA negativity at 3 months	0.750		
HBV DNA negativity at 6 months	0.834		
HBV DNA negativity at 12 months	0.681		
YMDD mutation	0.522		

LRE, liver-related event; CI, confidence interval; ALT, alanine aminotransferase; HBeAg, hepatitis B e antigen; kPa, kilopascal.

### Incidence of LREs according to fibrosis stage and LSM values

**Figure 3:**
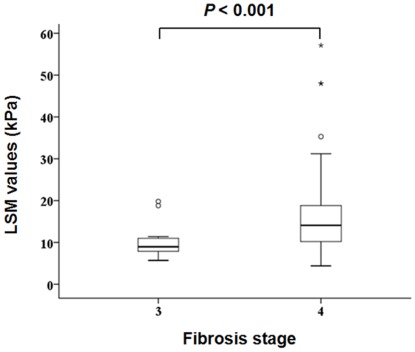
Box plots of LSM values in patients with F3 and F4 fibrosis stage. Median LSM value of patients with F3 and F4 were 9.0 (range, 5.7–19.8) kPa and 14.1 (range, 4.4–57.1) kPa, respectively and LSM values in patients with F4 fibrosis stage were significantly higher than those with F3 (10.1±3.7 *vs*. 15.8±8.8 kPa, *P*<0.001). LSM, liver stiffness measurement; kPa, kilopascal.

The median LSM values of patients with F3 and F4 fibrosis stages were 9.0 (5.7–19.8) kPa and 14.1 (4.4–57.1) kPa, respectively. LSM values were significantly higher in patients with F4 fibrosis stage than in those with F3 (10.1±3.7 *vs*. 15.8±8.8 kPa; *P*<0.001; **Figure 3**).

**Figure 4:**
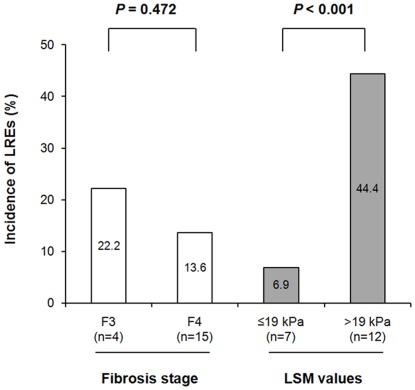
Incidence of LREs according to fibrosis stage and LSM values. The incidence of LREs was similar between patients with F3 fibrosis stage and those with F4 (22.2% *vs*. 13.6%, *P* = 0.472) whereas it was signficanly different between patients with LSM value ≤19 kPa and those with LSM value >19 kPa (6.9% *vs*. 44.4%, *P*<0.001). LRE, liver-related event; LSM, liver stiffness measurement; kPa, kilopascal.

The mean follow-up periods of patients with F3 and F4 fibrosis stages were similar (24.0 *vs*. 24.7 months; *P* = 0.827). The incidence of LREs was similar in patients with F3 and F4 fibrosis stages (4/18, 22.2% *vs*. 15/110, 13.6%; *P* = 0.472), whereas it differed significantly between patients with LSM values ≤19 kPa and those with LSM values >19 kPa (7/101, 6.9% *vs*. 12/27, 44.4%; *P*<0.001; **Figure 4**).

### Discordance between baseline LSM value and LRE development

As shown in **Figure 4**, discordant results between LSM values and LRE development were identified in 15/27 (55.6%) patients who did not experience LRE development despite baseline LSM values >19 kPa and 7/101 (6.9%) patients who developed LREs despite LSM values ≤19 kPa. However, no independent variable that could predict this discordance between LSM value and LRE development was identified.

### Influence of dynamic LSM changes on LRE development

With the exception of 14 patients without follow-up LSMs before LRE development, 114 patients underwent a second LSM before LRE development at a median interval of 13.1 (range, 3.8–51.6) months. Of these, LREs developed in 10 (8.8%) patients.

To estimate the LRE incidence according to LSM change, we stratified the patients into three groups as follows: baseline and follow-up LSM values ≤19 kPa (*n* = 91), baseline LSM >19 kPa and follow-up LSM ≤19 kPa (*n* = 11), and any baseline and follow-up LSM values >19 kPa (*n* = 12). The overall incidence of LRE development did not differ among groups (*P*>0.05). Although we further stratified the study population [LSM value increased by >30% of baseline LSM (*n* = 10), change in LSM values ≤30% of baseline LSM (*n* = 70), and LSM decreased by >30% of baseline LSM (*n* = 34)] [26]. no difference in LRE development was identified (*P*>0.05).

## Discussion

Advanced liver fibrosis or cirrhosis is significantly related to an increased risk of hepatic decompensation and HCC development, which, in turn, can worsen the prognosis of patients with CLD [27]. At a time when the natural course of chronic viral hepatitis could be observed due to the absence of antiviral agents, the incidence of HCC in highly endemic areas was approximately 1/100 person-years for CHB patients without cirrhosis [28], [29]. Other Asian studies reported that the incidence of HCC in untreated patients with compensated cirrhosis increased to 3–8/100 person-years [30], [31]. Moreover, the 5-year cumulative incidence of hepatic decompensation was reported as 16–20% (3.3–4/100 person-years) [32], [33]. In our study, the incidence of HCC and hepatic decompensation seemed relatively low (4.7/100 and 2.0/100 person-years, respectively), which can be explained in part by the relative short follow-up period and the inclusion of patients with F3 fibrosis stages.

However, effective antiviral agents such as NUCs and interferon (IFN) have emerged and are actively used to prevent or delay disease progression in patients with chronic viral hepatitis [34]–[36]. Hence, the natural course of chronic viral hepatitis has changed and some recent studies have demonstrated improved prognosis in such patients. George *et al.* [37] concluded that CHB patients receiving NUCs had a significantly lower incidence of HCC compared with untreated controls (2.4% *vs.* 6.4%). The regression of liver fibrosis due to the long-term use of antiviral agents may explain the improved long-term prognosis [38], [39]. However, because not all patients receiving antiviral treatment experience liver fibrosis regression [40], hepatic decompensation and HCC can eventually occur in some patients despite antiviral treatment.

Baseline HBV DNA level is the most important risk factor for HCC development without antiviral treatment [41]. However, we identified a significant predictive role for LSM with 19 kPa as an optimal cutoff value when appropriate suppression of HBV DNA using antiviral treatment was available. Similarly, Zakareya *et al.* identified a significant association between LSM and the development of cirrhosis-related complications in patients with CLD, and concluded that LSM values >32 kPa was associated with HCC development [42]. Because 19–32 kPa for predicting LRE development is much higher than the generally accepted cutoff LSM value for cirrhosis (10.3–11.0 kPa) [43], [44], it can be postulated that cirrhosis can be further sub-classified, which indicates that all patients with cirrhosis do not have identical prognoses according to severity.

Because our study enrolled only patients with available histology before starting antiviral treatment, our results cannot be directly applied to patients who will receive antiviral treatment without baseline LBs. However, the purpose of this study was to evaluate the additional clinical implications of LSM value, when compared with histologic data as a reference standard, for CHB patients before starting antiviral treatment using NUCs. We demonstrated that LSM with an optimal cutoff value might be useful in assessing the risk of LRE development in these patients, which is impossible using histologic data alone. Thus, LSM is not only a noninvasive tool for the one-time evaluation of the degree of liver fibrosis, but also a significant predictor of LRE development, which should be checked before antiviral treatment regardless of LB data, in CHB patients. Our results also suggest that LSM is more useful than LB, and that the incidence of LREs could only be identified using LSM, not histologic data. Since LSM values perform better than histology, further studies investigating the predictive ability of LSM in patients undergoing antiviral treatment using NUCs without baseline LB data are needed.

In our study, there was a significant overlap in LSM values between patients with F3 (5.7–19.8 kPa) and F4 (4.4–57.1 kPa), although the overall LSM values were significantly higher in F4 fibrosis stage. This can be explained in part by the overestimating influence of necroinflammatory activity on LSM [45]. Indeed, the proportion of high activity (A3–4) in F3 showed a trend to be higher than those of F4 (35.0% vs. 26.9%). Furthermore, LSM values of patients with F3 and high activity (A3–4) was statistically similar with those of patients with F4 and low activity (A1–2) (mean 12.7 vs. 14.6 kPa, *P*>0.05). All these results might have caused some overlapping values between patients with F3 and F4.

The prediction of LRE development using LSM was imperfect. Although patients with baseline LSM values >19 kPa were at a significantly greater risk of LRE development (HR, 7.176) than were those with baseline LSM values ≤19 kPa, 55.6% of patients with LSM values >19 kPa developed no LRE and 6.9% of those with LSM values ≤19 kPa did. In the sub-group analysis, we found no significant predictor of discordant results regarding LRE development. Because this finding might be related to statistical error due to the short-term follow-up period and the low number of LREs, large-scale studies with long-term follow-up are needed to elucidate a novel serological predictor of LRE development. When we increased the sensitivity of cutoff LSM value up to 89.5%, 9.1 kPa was selected. Using this cutoff value, we can clinically identify the sub-group of patients at low risk of LRE development (1.6%), such that these patients can be reassured.

LSM has been known to predict fibrosis regression in response to long-term antiviral treatment [46]. Thus, we further analyzed the role of LSM as a dynamic indicator of LRE development using cutoff LSM values of 19 kPa or relative change in LSM values from baseline, but the results were negative. However, small sample size of some groups (n = 11 and 12, respectively) might be related to type II error. Furthermore, because the study by Jung *et al*. [14] revealed that LSM change, similar to baseline LSM value, can influence HCC development, large cohorts with sufficient events will likely be required to investigate the usefulness of serial LSM value follow-up in patients with chronic viral hepatitis.

We included only patients with histologically advanced liver fibrosis (≥F3). However, because histologic evaluation grades liver fibrosis categorically, it cannot exactly represent the continuous spectrum of liver fibrosis, especially between adjacent fibrosis grades. Furthermore, because histologic evaluation of liver fibrosis can be influenced by intra- and interobserver variability, the over- or underestimation of liver fibrosis inevitably occurs. Considering these limitations of histologic evaluation, some patients who were excluded due to F0–F2 liver fibrosis may represent underestimations of F3 or F4 fibrosis, which might have resulted in selection bias, a major limitation of this study. Thus, further validation of LSM cutoff values for predicting advanced liver fibrosis regardless of histologic data should be performed. Furthermore, the significantly higher proportion of patients with F4 fibrosis may be another bias of this study.

In conclusion, our data suggest that LSM can be a useful predictor of LRE development in CHB patients showing histologically advanced liver fibrosis. However, this finding should be confirmed in CHB patients without baseline histologic data before the widespread application of LSM to all patients undergoing antiviral treatment.

## Supporting Information

Figure S1 **Recruitment algorithm.** A total of 178 consecutive chronic hepatitis B patients were enrolled. After 50 patients were excluded according to our exclusion criteria, a total of 128 patients were selected for statistical analysis. CHB, chronic hepatitis B; LSM, liver stiffness measurement; LB, liver biopsy; HCC, hepatocellular carcinoma.
(TIF)
