# Supplementary figures and images for: Prediction of Liver-Related Events Using Fibroscan in Chronic Hepatitis B Patients Showing Advanced Liver Fibrosis
Source: PLoS One. 2012 May 4;7(5):e36676. doi: 10.1371/journal.pone.0036676 (PMC3344942; doi:10.1371/journal.pone.0036676)

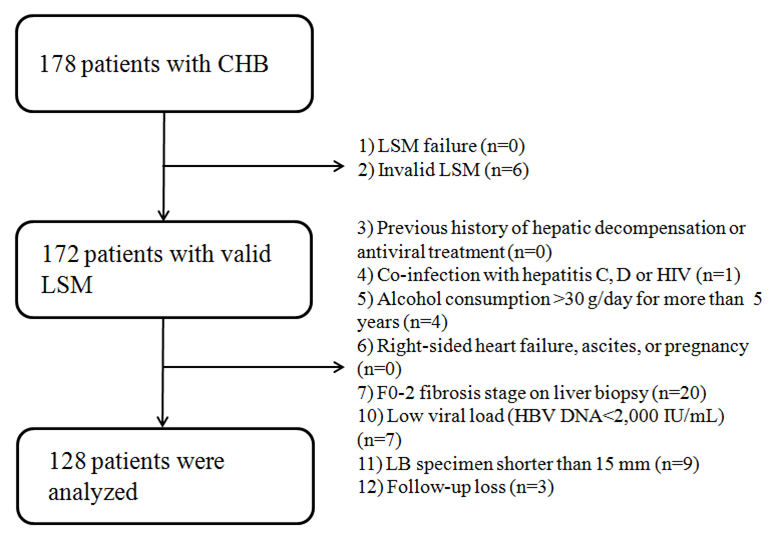

Supplement: Figure S1 — Recruitment algorithm. A total of 178 consecutive chronic hepatitis B patients were enrolled. After 50 patients were excluded according to our exclusion criteria, a total of 128 patients were selected for statistical analysis. CHB, chronic hepatitis B; LSM, liver stiffness measurement; LB, liver biopsy; HCC, hepatocellular carcinoma. (TIF) [file pone.0036676.s001.tif]
